# Pesticide Residues in Mandarins: Three-Year Monitoring Results

**DOI:** 10.3390/molecules28145611

**Published:** 2023-07-24

**Authors:** Emrah Gormez, Ozgur Golge, Miguel Ángel González-Curbelo, Bulent Kabak

**Affiliations:** 1 Pia Frucht Food Control Laboratory, Alaşehir 45600, Turkey; 2 Department of Gastronomy and Culinary Arts, Faculty of Tourism, Alanya Alaaddin Keykubat University, Alanya 07425, Turkey; 3 Departamento de Ciencias Básicas, Facultad de Ingeniería, Universidad EAN, Calle 79 n° 11-45, Bogotá 110221, Colombia; 4 Department of Food Engineering, Faculty of Engineering, Hitit University, Corum 19030, Turkey; 5 Biotechnology Laboratory, Machinery and Manufacturing Technology Application and Research Center, Hitit University, Corum 19030, Turkey

**Keywords:** chromatography, food safety, mass spectrometry, pesticides, polar pesticides, QuEChERS, QuPPe

## Abstract

The demand of plant production product use has increased because of the current system of citrus production, which prioritizes high agricultural yields. Therefore, the monitoring of pesticide residues in citrus fruits and other agricultural products and their impacts on human health and food security are of great concern. This study aims to determine multi-class pesticides including highly polar residues in satsuma mandarins. A total of 226 mandarin samples were collected over three consecutive harvesting years from 2019 to 2021 in the Izmir region of Turkey. Targeted compounds included pesticides and metabolites with European Union (EU) regulatory levels, plus other non-approved residues and highly polar compounds. The residues excluding highly polar substances were analyzed by applying the quick, easy, cheap, effective, rugged, and safe (QuEChERS) extraction and liquid chromatography–triple quadrupole mass spectrometry (LC-MS/MS) determination for 434 analytes and gas chromatography–triple quadrupole mass spectrometry (GC-MS/MS) determination for 71 analytes. For six highly polar pesticides, sample preparation was based on Quick Polar Pesticides (QuPPe) extraction. The polar residues were determined by LC-MS/MS using internal standards. Forty different residues, including two highly polar substances, were recorded in mandarin samples through three harvesting years. In 8.4% of the samples, no quantifiable residues were detected, whereas 207 samples contained at least one residue. The maximum residue level (MRL) exceedances were recorded for 22.1% of the samples. The two most frequently found pesticides were phosphonic acid and spirotetramat, with an incidence rate of 48.7% and 46.5%, respectively. The concentration of phosphonic acid and spirotetramat in mandarin samples varied from 0.026 to 39.386 mg kg^−1^ and from 0.010 to 1.485 mg kg^−1^, respectively. The results will enable researchers and regulatory authorities to assess the extent of pesticide presence, identify potential risks, and take necessary measures to ensure the safety of satsuma mandarins for consumers.

## 1. Introduction

Mandarins, also known as tangerines in some parts of the world, are the second most commonly cultivated citrus type, with 38 million tons (22.4% of global citrus production), after oranges but ahead of lemons and grapefruit. In the 2020 season, the global mandarin crop totaled over 38 million tons. China is the global supply leader with over 23 million metric tons, accounting for more than 60% of the world’s mandarin crops in 2020/2021. Spain and Turkey came in second and third, with 5.6% and 4.1% of the global market share, respectively. In 2020, Spain was the top exporter of mandarins with over 1.3 million tons, accounting for 23.4% of the global exports. Turkey and China were the second- and third-largest exporters of mandarins, accounting for 15% and 12.8% of the global market, respectively. In 2020, the Russian Federation was the leading importer of mandarins in the world, with a 16.9% share of global imports, followed by the United States (7.4%), Germany (7.3%), France (6.7%), and the United Kingdom (5.9%) [1].

Carbohydrates, mainly sucrose, glucose, and fructose, and dietary fiber are the principal macronutrients in mandarins. They are also a well-known source of many valuable substances such as organic acids (mainly citric and malic acids), carotenoids (β-cryptoxanthin), polyphenols (flavonoids and phenolic acids), vitamin C, and minerals (mainly potassium) [2]. When compared to other citrus fruits such as lemons, oranges, or grapefruits, mandarins are generally not suited to long-term storage.

Many different pathogens, including insect pests such as the Mediterranean fruit fly (*Ceratitis capitata*), affect mandarins and other citrus fruits, causing diseases with adverse effects in orchards worldwide. Mandarins are susceptible to various citrus diseases, including citrus blast caused by the bacterial pathogen *Pseudomonas syringae*, citrus canker caused by *Xanthomonas* spp., anthracnose (*Colletotrichum* spp.), green mold caused by *Penicillium digitatum*, blue mold caused by *Penicillium italicum*, collar rot caused by *Phytophthora citrophthora,* sour rot caused by *Geotrichum citri-aurantii*, *Alternaria* brown spot caused by *Alternaria* spp., gray mold caused by *Botrytis cinerea*, and *Mucor* rot caused by *Mucor piriformis* [3,4].

In spite of the rising consumer resistance to the presence of chemical residues on products, the utilization of pesticides remains the prevailing practice for the prevention of pre-harvest and post-harvest infestations. Very small amounts of pesticides called residues may remain in or on fruits and vegetables and might pose a potential risk to human health due to their sub-acute and long-term toxicity. For this reason, it is very important to control and regulate pesticide use in agricultural production and to monitor their levels in fruits and vegetables [5]. National and international organizations establish a maximum residue level (MRL) for each agricultural and other product, aiming to establish benchmarks for food safety and promote global trade. In Turkey, the Ministry of Agriculture and Forestry bears the responsibility of assessing the permissible levels of pesticide residues in agricultural and other products [6], adhering to the regulations set forth by European Union (EU) legislation [7], to ascertain the levels of residues.

Pesticides are widely used in fruit growing and in the treatment of citrus fruits for pre-harvest and post-harvest protection by the citrus farmers in Turkey and around the world. The Commission’s Rapid Alert System for Food and Feed (RASFF) shows that in 2022, a total of 293 notifications on fruits and vegetables from Turkey were transmitted through the system, 23 of which (7.85%) concerning mandarins [8]. Following an increase in the number of interceptions of Turkish citrus fruits that do not meet requirements on pesticide residues, the European Commission has decided to temporarily increase by 20% the frequency of physical checks on citrus fruits, including mandarin and clementine imports from Turkey [9].

Methods used for the analysis of pesticides vary widely. However, liquid chromatography and gas chromatography coupled with triple quadrupole mass spectrometry (LC-MS/MS and GC-MS/MS) methods are the most powerful techniques in the determination of pesticides [10,11,12,13]. While there are many sample-extraction methods including solid-phase extraction [14,15], solid-phase microextraction [16], liquid–liquid extraction [17], liquid-phase microextraction [18], pressurized liquid extraction [12], accelerated solvent extraction [19], ultrasonic solvent extraction [12], supercritical fluid extraction [20,21], ultrasonic solvent extraction [12], matrix solid-phase dispersion [22,23], and microwave-assisted extraction [24], the quick, easy, cheap, effective, rugged, and safe (QuEChERS) extraction method developed by Anastassiades et al. [25] has increasingly being used in combination with LC-MS/MS and/or GC-MS/MS for the detection of multi-class residues in agricultural and other products. QuEChERS has gained significant popularity in pesticide residue analysis due to its simplicity, time saving, cost-effectiveness, high throughput, and minimal solvent requirement. In the QuEChERS extraction method, the process comprises two steps: extraction and clean-up. In the first step, the residues are extracted from the matrix with acidified acetonitrile and salts/buffers. To reduce interferences, sugars, fatty acids, organic acids, lipids, and polar pigments are removed in the clean-up step by the use of primary–secondary amine (PSA). However, highly polar pesticides have been excluded for a long time from the routine scope of laboratory investigations because they are not amenable to extraction via QuEChERS. Recently, the Quick Polar Pesticides (QuPPe) extraction method for the simultaneous analysis of highly polar substances has been developed by the EU Reference Laboratory for Pesticides Single Residue Methods (EURL-SRM). With this technique, many polar substances are extracted with acidified methanol from the various matrices without a sample clean-up process [26].

The main purpose of this study was to monitor the residual concentration of pesticides in Turkish satsuma mandarins (*Citrus unshiu* Marcovitch) intended for export during three harvesting years. The methodologies involved the QuEChERS and QuPPe sample preparation approaches for the determination of 505 non-polar/medium-polar and six highly polar residues, respectively. The QuEChERS extracts were analyzed by LC-MS/MS and GC-MS/MS, whereas only LC-MS/MS in electrospray negative ionization (ESI-) mode was used in the determination stage for QuPPe extracts.

## 2. Results and Discussion

### 2.1. Method Validation Data

In-house method validation involving LC-MS/MS and GC-MS/MS was conducted to establish method performance characteristics for the detection and quantification of target compounds in the matrix of high acid content and high water content. The method validation data for the detected residues are shown in Table S1. The LC-MS/MS and GC-MS/MS methods demonstrated satisfactory selectivity. No visible interfering peaks were evident at or close to the expected retention times of the target analytes. Linearity of response was acceptable (coefficient of determination (*R*^2^) > 0.99) for the majority of the compounds (except for five residues: dinobuton, hexachlorobenzene, tefluthrin, tralkoxydim, and vinclozolin) including highly polar substances. Method limits of quantification (LOQ) for all target compounds were lower than 0.01 mg kg^−1^. All recovery values are compliant with provisions set in SANTE 11312/2021 Guideline [27], which recommends a recovery rate of 70–120%. A recovery range of 73.9 to 113.5% was observed after a spiking matrix with detected analytes at 0.01 mg kg^−1^. After extraction of the higher level compound-spiked matrix, recoveries of detected residues fell within the range of 86.5 to 109.9%. For the blank matrix spiked with residues at 0.01 and 0.05 mg kg^−1^, the repeatability (RSD_r_, %) was found to be from 0.20 to 16.31% and from 0.27 to 8.41%, respectively, for detected residues. The within-laboratory method reproducibility (RSD_R_) data were found to be in the range of 0.62 to 13.56% and 1.73 to 6.65% at 0.01 and 0.05 mg kg^−1^ spiking levels, respectively. The measurement uncertainties for detected residues were between 8.5 and 42.8%.

### 2.2. Pesticide Residues in Mandarins

Between 2019 and 2021, a total of 226 mandarin samples cultivated in the Izmir region, Turkey, were monitored for the presence of 511 pesticide residues. Monitoring pesticide residues over multiple years provides a more robust and representative dataset, as it helps account for potential variations in pesticide use and fruit quality across different harvest seasons. Agricultural practices, including pesticide application, can vary from year to year based on factors such as weather conditions, pest pressure, and farmer practices. By sampling satsuma mandarins over three years, the study can better capture the overall trend and consistency of pesticide contamination in the region.

At least one pesticide residue was detected in 91.6% of the analyzed samples, while no pesticide residue was found in 19 samples. Forty different pesticides were detected in the mandarin samples, including 23 insecticides, 14 fungicides, two acaricides, and one insect growth regulator. Among the 40 active substances recorded in mandarin samples, 12 of them were non-approved in the EU. Only fosetyl and phosphonic acid were detected in mandarins among the six highly polar residues (chlorate, ethephon, fosetyl, glyphosate, perchlorate, and phosphonic acid).

In 2019, 29 mandarin samples were analyzed. Table 1 shows the quantified pesticide residues and their concentrations. Only three samples (10.3%) were free of quantifiable residues. In total, 89.7% of mandarin samples contained at least one pesticide residue, but only two of them (6.9%) exceeded the MRL. These two exceedances were related to residues of buprofezin and propiconazole.

**Figure 1:**
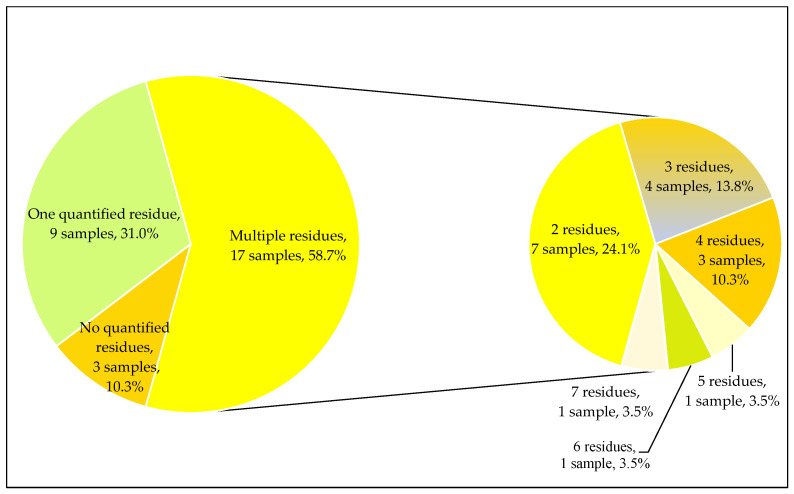
Number of quantified residues in satsuma mandarins from 2019.

In mandarin samples from 2019, 22 different residues were detected in quantifiable concentrations. While the majority of recorded residues (16 pesticides) relate to approved pesticides, six non-approved pesticides (carbendazim, chlorpyrifos-methyl, imidacloprid, phosmet, propiconazole, and thiophanate-methyl) were found in different mandarin samples. In total, 31% of mandarin samples contained only one residue, while multiple residues were quantified in 17 samples (58.7%); mandarin samples were recorded with up to seven different residues (Figure 1).

The most frequent residue detected in mandarins from 2019 was phosphonic acid, with a detection rate of 72.4%. The presence of this compound can be attributed to the utilization of fungicides such as fosetyl and phosphonic acid salts, as well as the prior application of growth enhancers. Notably, phosphonic acid is encompassed within the permissible MRL for fosetyl-aluminium (fosetyl-Al), considering the cumulative amount of fosetyl, phosphonic acids, and their respective salts, calculated as fosetyl [28]. The concentration of phosphonic acid varied from 0.028 to 3.835 mg kg^−1^ (0.038–5.49 mg kg^−1^ for fosetyl, sum), with a mean level of 0.826 mg kg^−1^ (1.12 mg kg^−1^ for fosetyl, sum). Only two mandarin samples from 2019 contained fosetyl per se in concentrations of 0.006 and 0.351 mg kg^−1^.

Residues of malathion (34.5%; range = 0.014–0.716 mg kg^−1^), spirotetramat (17.2%; 0.017–0.112 mg kg^−1^), sulfoxaflor (17.2%; 0.010–0.015 mg kg^−1^), acetamiprid (10.3%; 0.010–0.129 mg kg^−1^), and tau-fluvalinate (10.3%; 0.063–0.228 mg kg^−1^) were found in more than 10% of the mandarin samples. While lambda-cyhalothrin was detected in two mandarin samples (0.029–0.190 mg kg^−1^) from 2019 in measurable concentrations, fourteen pesticides, namely buprofezin (0.013 mg kg^−1^), carbendazim (0.038 mg kg^−1^), chlorpyrifos-methyl (0.010 mg kg^−1^), deltamethrin (0.015 mg kg^−1^), fludioxonil (0.627 mg kg^−1^), fluopyram (0.013 mg kg^−1^), imazalil (0.801 mg kg^−1^), imidacloprid (0.012 mg kg^−1^), phosmet (0.014 mg kg^−1^), pirimicarb (0.037 mg kg^−1^), propiconazole (1.008 mg kg^−1^), pyrimethanil (0.701 mg kg^−1^), pyriproxyfen (0.028 mg kg^−1^), and thiophanate-methyl (0.022 mg kg^−1^), were quantified only in one sample.

In 2020, 93 mandarin samples were analyzed. Table 2 shows the quantified pesticide residues and their concentrations in mandarin samples in 2020. The quantification rate of pesticides in mandarin samples from 2020 (95.7%) was slightly increased compared to the 2019 results (89.7%). In 4.3% of mandarin samples, no measurable residues were recorded. A total of 67 mandarin samples (72%) contained at least one detectable pesticide within the legally permitted concentrations, whereas the MRL exceedances were recorded in 21 samples (22.6% of the analyzed samples in 2020). Compared with 2019, the MRL exceedance rate for mandarin went up. Residues exceeding the MRL were related to five residues (buprofezin, chlorpyrifos, fenbutatin-oxide, malathion, and propiconazole).

**Figure 2:**
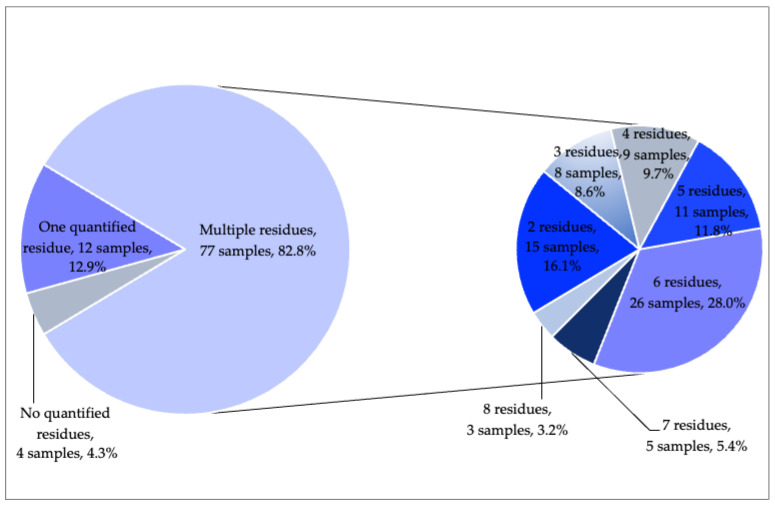
Number of quantified residues in satsuma mandarins from 2020.

In the 2020 monitoring year, 24 different residues were found at measurable concentrations in mandarin samples. While 20 detected residues in mandarins were approved pesticides, the remaining four residues (propiconazole, fenbutatin-oxide, spirodiclofen, and chlorpyrifos) were non-approved. While 12 mandarin samples contained only one residue, multiple residues were detected in 82.8% (77 samples) of the samples; up to eight pesticides were found in individual mandarin samples (Figure 2). It should be noted that the multiple-residue rate significantly increased from 55.2% in 2019 to 82.8% in 2020.

Among the residues, phosphonic acid (58.1%, range = 0.039–39.386 mg kg^−1^; 0.052–52.777 mg kg^−1^ for fosetyl, sum), spirotetramat (55.9%, 0.011–0.324 mg kg^−1^), fludioxonil (46.2%, 0.011–0.648 mg kg^−1^), imazalil (46.2%, 0.408–1.006 mg kg^−1^), pyrimethanil (46.2%, 0.329–1.200 mg kg^−1^), and 2-phenylphenol (44.1%, 0.584–2.667 mg kg^−1^) were the most frequently detected pesticides present in more than 40% of the mandarin samples. Compared to the 2019 results, the quantification rate was more than tenfold higher for the pesticides fludioxonil, imazalil, and pyrimethanil. The fungicide 2-phenylphenol, which was frequently detected in mandarins in 2020, was not recorded in 2019.

In 2021, 104 mandarin samples were analyzed. Table 3 shows the distribution of pesticide residue contents in mandarin samples from 2021. In 12 samples (11.54%) no pesticide residues were quantified, whereas 92 samples contained one or several pesticides in measurable concentrations. For 27 samples (25.96% of the analyzed mandarin samples), the residue concentrations exceeded the MRL. These exceedances were mainly related to buprofezin residue (16 samples), followed by propiconazole (7 samples), fenbutatin-oxide (5 samples), and spirotetramat (4 samples). Among the 39 individual determinations that exceeded the MRL, 18 determinations were observed for residues that are currently non-approved.

**Figure 3:**
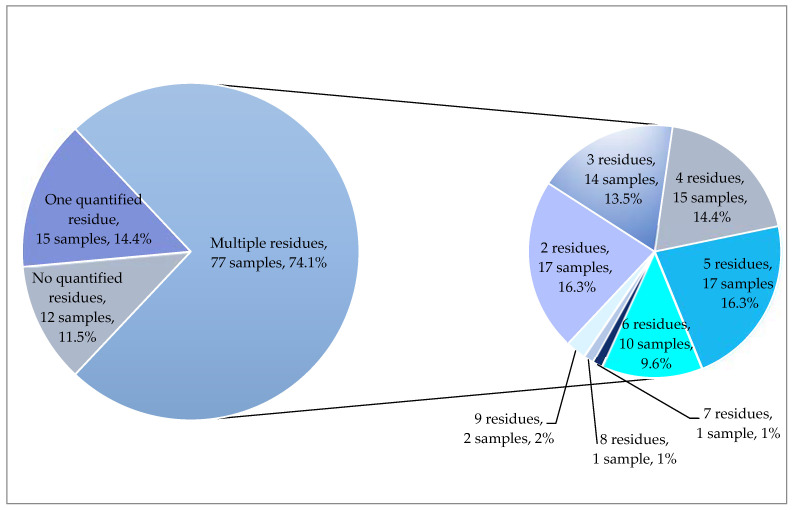
Number of quantified residues in satsuma mandarins from 2021.

Compared with 2019 and 2020, a higher number of residues were found in mandarins in 2021. In total, 32 different pesticides were recorded in concentrations equal to or above the LOQ in mandarin samples from 2021. In 14.4% of the samples, only one residue was found in quantifiable concentrations. Multiple residues were recorded in 74.1% of the samples; up to nine residues were detected in individual mandarin samples from 2021 (Figure 3). Among the 77 samples that contained more than one residue, 22.1% of which (17 samples) had two residues, 18.2% (14 samples) three residues, 19.5% (15 samples) four residues, 22.1% (17 samples) five residues, 13% (10 samples) six residues, 1.3% (one sample) seven residues, 1.3% (one sample) eight residues, and 2.6% (two samples) nine residues.

The most frequently detected pesticide was spirotetramat in mandarin samples from 2021, with an incidence rate of 46.2% (48 samples). The samples contained spirotetramat concentrations ranging from 0.010 up to 1.485 mg kg^−1^, with a mean concentration of 0.155 mg kg^−1^. The MRL of 0.5 mg kg^−1^ for spirotetramat was exceeded for only four mandarin samples. The insecticide spirotetramat, derived from tetramic acid, has been widely used in citrus orchards in Turkey for the control of sucking insects, including *Planococcus citri*, *Aonidiella citrina*, *Aonidiella aurantia*, *Aphis gossypi*, and *Aphis citricola* [29]. It acts as an acetyl-coA carboxylase inhibitor and interrupts the biosynthesis of lipids in insects. After the foliar application of spirotetramat, it enters the plant and transforms into its metabolite enol, along with the metabolites -enol-glucoside and -ketohydroxy, which are the three main products of degradation [30,31]. Its derivatives are included in the current MRL for spirotetramat (sum of spirotetramat and their derivatives, spirotetramat-enol, spirotetramat-enol-glucoside, spitotetramet-monohydroxy, and spirotetramat-ketohydroxy, calculated as spirotetramat, sum) [32]. For spirotetramat, an acceptable daily intake (ADI) of 0.05 mg kg^−1^ body weight (b.w.) day^−1^ and an acute reference (ARfD) dose of 1 mg kg^−1^ b.w. have been set [31].

The second most frequently detected residue in mandarin samples from 2021 was phosphonic acid. This residue was recorded in 33.7% of samples (35 samples) at levels ranging from 0.026 to 5.342 mg kg^−1^ (0.035–7.158 mg kg^−1^ for fosetyl, sum) with a mean concentration of 1.844 mg kg^−1^ (2.497 mg kg^−1^ for fosetyl, sum). Moreover, six mandarin samples contained fosetyl per se in amounts up to 0.164 mg kg^−1^. None of the samples exceeded the MRL of 150 mg kg^−1^ for fosetyl, sum.

Acetamiprid was also found commonly in mandarin samples with an occurrence value of 25% (26 samples), but all of them were far below the EU MRL of 0.9 mg kg^−1^. The concentration of acetamiprid in samples varied from 0.010 to 0.121 mg kg^−1^ (mean = 0.032 mg kg^−1^). The residues quantified in more than 10% of the mandarin samples from 2021 were pyriproxyfen (21.2%, 22 samples), tau-fluvalinate (20.2%, 21 samples), malathion (19.2%, 20 samples), buprofezin (15.4%, 16 samples), spirodiclofen (14.4%, 15 samples), pyridaben (13.5%, 14 samples), fludioxinil (12.5%, 13 samples), 2-phenyl phenol (11.5%, 12 samples), pyrimethanil (11.5%, 12 samples), and imazalil (10.6%, 11 samples). The 18 other residues were found in less than 10% of the samples, nine of them were non-approved pesticides (propiconazole, fenbutatin-oxide, chlorpyrifos, imidacloprid, thiacloprid, novaluron, bifenthrin, chlorpyrifos-methyl, and thiophanate-methyl). The MRL was exceeded for nine pesticides: buprofezin (sixteen samples), propiconazole (seven samples), fenbutatin-oxide (five samples), spirotetramat (four samples), novaluron (two samples), thiacloprid (two samples), chlorpyrifos (one sample), chlorpyrifos-methyl (one sample), and pyridaben (one sample).

In a previous study, 38 out of 70 mandarin samples (54.3%) collected from the Izmir and Mugla regions of Turkey contained at least one residue. Imazalil was found to be the most frequently recorded residue, with a level of 0.024–0.494 mg kg^−1^ [33]. In 2010–2012, 29 mandarin samples collected from a market in the Aegean region of Turkey were screened for the presence of 186 pesticides. In total, 83% of mandarin samples contained at least one residue, while MRL exceedance was recorded in only one sample. Nine different residues were detected in mandarin samples. Chlorpyrifos (34.5%, 0.01–0.226 mg kg^−1^), dimethomorph (31%, 0.019–0.062 mg kg^−1^), imazalil (24.1%, 0.933–2.47 mg kg^−1^), pyriproxyfen (24.1%, 0.01–0.065 mg kg^−1^), and malathion (20.7%, 0.03–1.01 mg kg^−1^) were reported to be the most frequently found residues in mandarin samples [34].

In a recent study by Al-Nasir et al. [35], citrus fruits cultivated at three locations in the Jordan Valley were monitored for 304 pesticides. Five residues, namely chlorothalonil (100%, 6.607–16.867 mg kg^−1^), chlorsulfuron (100%, 0.033–0.127 mg kg^−1^), iodosulfuron-methyl (100% 0.042–0.125 mg kg^−1^), bensulfuron-methyl (80%, 0.028–0.049 mg kg^−1^), and daminozide (80%, 0.056–0.920 mg kg^−1^) were recorded in most of the mandarin samples, with a detectable frequency ranging from 80% to 100%. In a Chinese survey from 2013 to 2018, 2922 citrus samples (1227 orange samples and 1695 mandarin/tangerine samples) were monitored for the presence of 106 targeted banned or commonly used pesticides. Forty different pesticides including 20 insecticides, 14 fungicides, and 6 acaricides were found in citrus samples. The three most frequently detected residues in citrus fruits were reported to be chlorpyrifos (40%, 0.020–0.90 mg kg^−1^), prochloraz (26%, 0.005–3.7 mg kg^−1^), and carbendazim (21%, 0.005–1.9 mg kg^−1^) [36]. In the 2015 official control activities of EU member states, Iceland, and Ireland, 79.6% of 1331 mandarin samples were reported to contain at least one residue, while multiple residues were found in 63.6% of samples (*n* = 846) [37]. It should also be noted that the pesticides such as bensulfuron-methyl, chlorothalonil, daminozide, dimethomorph, iodosulfuron-methyl, and prochloraz detected in mandarins according to previous studies were monitored in the present study, but they were not detected in the samples throughout the three years.

Although valuable findings were presented, the current study has a few limitations. A larger sample size and a more diverse geographical distribution could improve the representativeness of the results. The study focused on quantifying pesticides and comparing them to MRLs without conducting a risk assessment and toxicological analysis. The potential cumulative effects or interactions of multiple residues were not addressed. The study considers pesticide residues in whole fruits but does not account for potential pesticide degradation or loss during post-harvest handling and processing such as washing, peeling, and juicing.

## 3. Materials and Methods

### 3.1. Chemicals, Reagents, and Standards

Acetonitrile and methanol used for the preparation of calibration standards, spiking solutions, sample extraction, and mobile phases for LC separation were LC-MS grade (J.T. Baker, Gliwice, Poland). Mobile phase modifiers including ammonium formate and glacial acetic acid, and formic acid were of analytical grade (Merck KGaA, Darmstadt, Germany). QuEChERS extraction kits were supplied from Agilent. Deionized water was obtained using a Milli Q (Millipore, Molsheim, France) Direct Q3 water purification system.

Individual pesticide standards were purchased from Dr. Ehrenstorfer GmbH (Augsburg, Germany). Triphenyl phosphate (TPP, internal standard) and isotopically labeled internal standards (ILISs) of etephon D4, and fosetyl-Al D15 were supplied from Dr. Ehrenstorfer GmbH (Augsburg, Germany). The ILIS of ^18^O_3_-phosphonic was obtained from Toronto Research Chemicals (Toronto, ON, Canada). The purity of the ILISs was >94%.

### 3.2. Samples

A total of 226 satsuma mandarin samples, each weighing 2 kg, were collected from Izmir province, Turkey, for the analysis of 511 pesticide residues. Sampling was conducted over three consecutive years, from 2019 to 2021, with the number of samples ranging from 29 to 104 per year. The collection process followed the guidelines provided by the Commission Directive 2002/63/EC [38]. The samples were stored in cool conditions to maintain their freshness, specifically at 4 ± 1 °C, for no longer than two days. Mandarin samples were analyzed as sold without any processing. Prior to homogenization, the stem portion of the unwashed mandarin samples was removed. Mandarins were divided into four quarters with the peel intact, and the two diagonal segments were included in the homogenization process. Mandarin samples were homogenized using a laboratory food processor (Retsch GmBh, GM 300, Haan, Germany) to achieve consistent and small particle sizes. Each analytical result was derived from a single laboratory sample taken from each lot.

### 3.3. Sample Preparation

**Figure 4:**
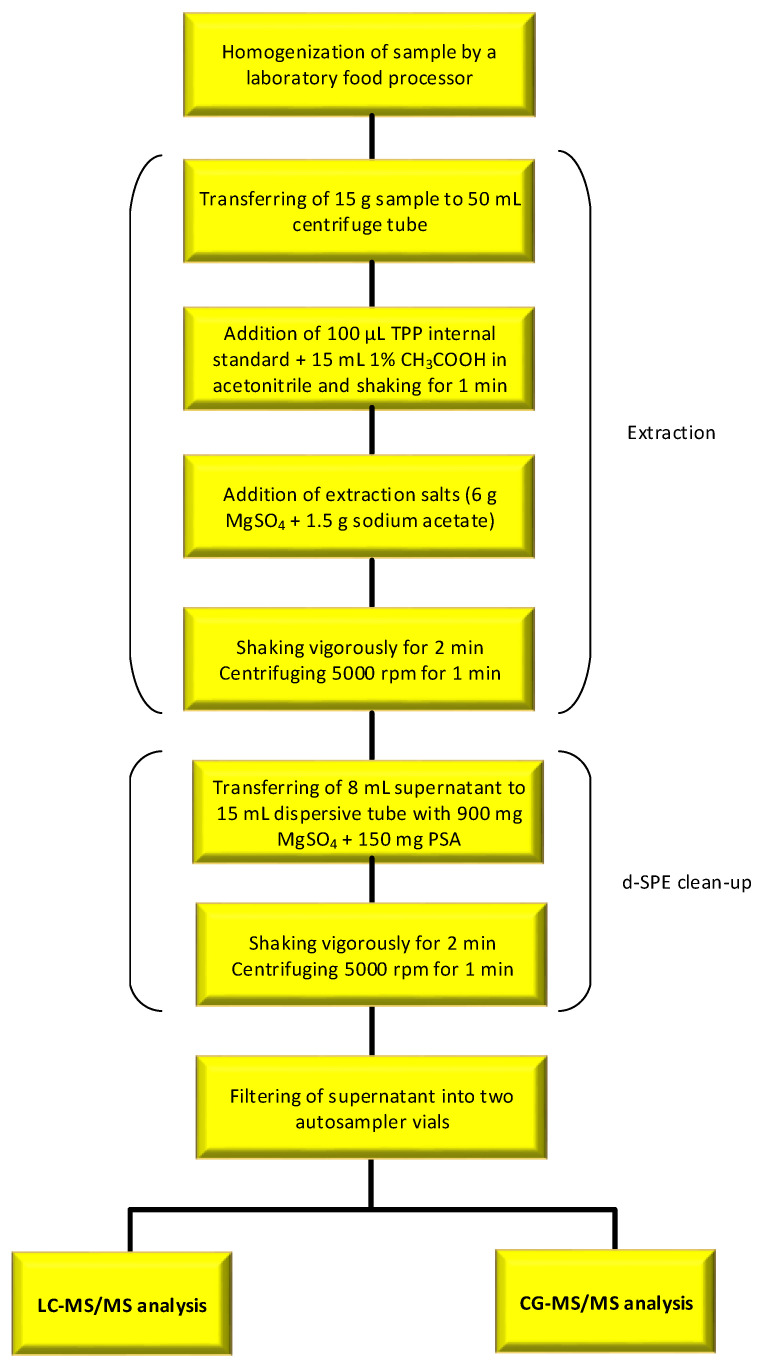
The schematic diagram of the QuEChERS sample preparation.

For extraction of multi-class pesticide residues except for polar pesticides, the Association of Official Analytical Chemists (AOAC) version of the QuEChERS method [39] was used with slight modifications. The QuEChERS sample preparation methodology was summarized in Figure 4. Briefly, 15 g of homogenized mandarin sample was placed into a 50 mL polypropylene extraction tube, and 100 μL of TPP solution (internal standard, 10 μg mL^−1^) and 15 mL of acetonitrile containing 1% acetic acid were added. After shaking the tube vigorously for 1 min, the QuEChERS salt extraction packet (containing 6 g of MgSO_4_ and 1.5 g of sodium acetate) for AOAC 2007 method was added. The tube was shaken on a platform shaker (Collomix GmbH, VIBA 330, Gaimersheim, Germany) for 2 min and centrifuged (Hettich, Rotofix 32A, Tuttlingen, Germany) for 1 min at 5000 rpm. After extraction, 8 mL of acetonitrile layer (supernatant) was transferred to a clean-up dispersive tube containing 900 mg MgSO_4_ and 150 mg PSA to remove residual water and further remove matrix interferences (sugars, organic acids, and polar pigments) from the sample. The tube was shaken on a platform shaker for 2 min and centrifuged (4000 rpm, 3 min). The supernatant was then filtered using a 0.20 μm cellulose syringe filter and analyzed by LC-MS/MS or GC-MS/MS.

**Figure 5:**
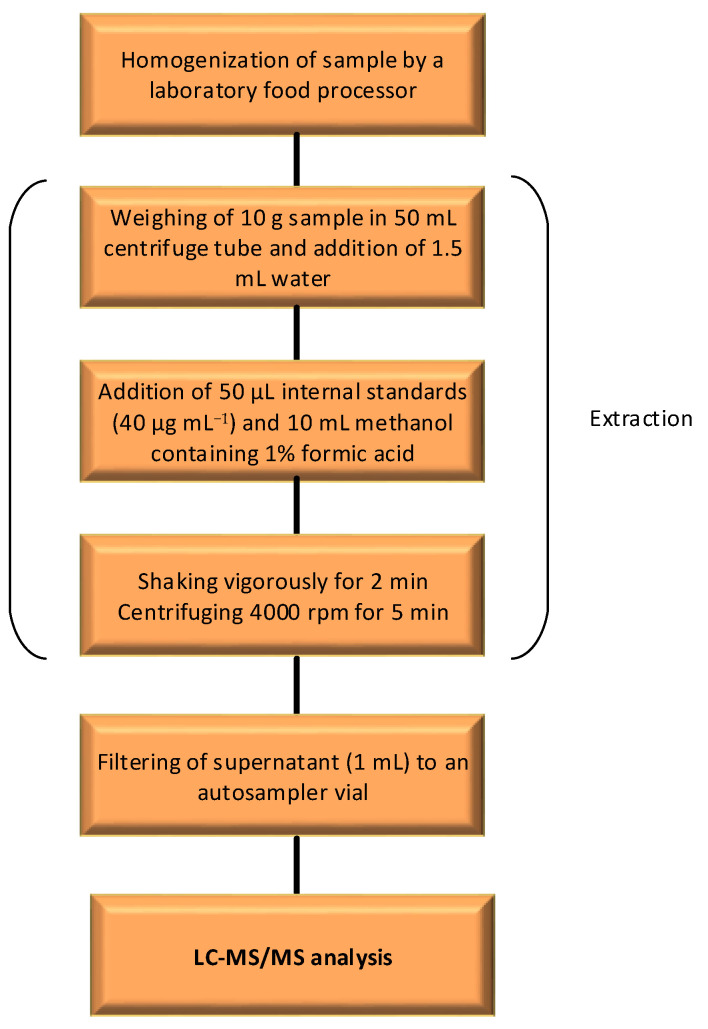
Workflow diagram for QuPPe sample preparation.

For extraction of six highly polar pesticides (chlorate, ethephon, fosetyl, glyphosate, perchlorate, and phosphonic acid) from mandarin samples, the QuPPe method developed by the EURL-SRM [26] was employed as shown in Figure 5. For mandarins, 10 g of homogenized sample was taken in a 50 mL centrifuge tube, and 1.5 mL of ultrapure water was added to adjust the total extract volume. Before the extraction with 10 mL of acidified methanol (containing 1% formic acid), 50 μL of ILISs solution (40 μg mL^−1^) was added to the tube. After shaking and centrifugation (4000 rpm for 3 min) steps, the methanol layer (supernatant) from the QuPPe extract was filtered through a 0.20 μm cellulose syringe filter and analyzed by LC-MS/MS.

### 3.4. LC-MS/MS Analysis

LC-amenable of 440 pesticides separation was conducted using an Agilent 1290 HPLC system (Agilent, Santa Clara, CA, USA). This was equipped with an autosampler, a degasser module, a binary pump, and temperature-controlled column oven. Via a jet stream electrospray ionization (ESI) source, the LC was coupled to an Agilent 6470 QQQ triple quadrupole mass spectrometer (MS/MS, Agilent, Santa Clara, CA, USA).

Chromatographic separation of LC-amenable pesticides (434 substances) except for polar substances was achieved using an InfinityLab Poroshell 120 SB-C18 column (3 × 100 mm, 2.7 μm particle size) (Agilent Technologies, Santa Clara, CA, USA). The column temperature was set at 45 °C, and the flow rate was set at 0.66 mL min^−1^. Eluent A was water, containing 5 mM ammonium formate, and eluent B was 100% methanol. Gradient elution was applied as follows: 0–0.5 min 40% B, 0.5–3.5 min: 40–60% B, 3.5–7 min: 60–98% B, 7–8.7 min 98% B, 8.7–8.8 min: 98–40% B, and 8.8–11 min: 40% B.

A porous graphitic carbon-based Hypercarb 2.1 × 100 mm column with 5 μm particle size (Thermo Scientific™, Waltham, MA, USA) was used for the separation of six polar compounds at 40 °C. The mobile phase for QuPPe extracts was composed of 94:5:1 water-methanol-acetic acid (*v*/*v*/*v*) as eluent A and methanol-acetic acid at the ratio of 99:1 (*v*/*v*) as eluent B. The gradient started at 0 min, 0% B, and increased linearly to 30% B in 10 min at a flow rate of 0.2 mL min^−1^. The 30% B was kept for 8 min and then increased linearly to 90% B in 1 min at a flow rate of 0.4 mL min^−1^. The 90% B was kept for 3 min and returned to 0% B within 0.1 min at the initial flow rate of 0.2 mL min^−1^ and held for 10 min.

Electrospray negative ionization (ESI-) was used for the analysis of QuPPe extract. The ionization conditions of the ESI source were as flows: gas temperature of 230 °C, gas flow of 10 L min^−1^, nebulizing gas pressure of 45 psi, sheath gas temperature of 300 °C, sheath gas flow of 11 L min^−1^, capillary of 3500 V, and nozzle voltage of 500 V. Nitrogen was used as the collision gas. Data acquisition was performed using Agilent MassHunter software (Version B.07.01).

### 3.5. GC-MS/MS Analysis

A total of 71 GC-amenable pesticides were analyzed using an Agilent (Santa Clara, CA, USA) 7890A GC system equipped with an Agilent 7693 autosampler, interfaced to an Agilent 7000B triple quadrupole mass spectrometer. An Agilent HP-5MS Ultra Inert analytical column (30 × 0.25 mm, 0.25 μm) was used in the residue separation, with helium as a carrier gas at a constant flow rate of 1.25 mL min^−1^. The GC oven was operated under the following conditions: initial temperature of 75 °C held for 2.5 min, 50 °C min^−1^ rate to 150 °C, then 20 °C min^−1^ rate to 200 °C, and finally 16 °C min^−1^ rate to 310 °C and held for 15 min. The injection port temperature was 280 °C and 5 μL volume was injected with a multimode inlet in programmable temperature vaporizer (PTV) mode.

The triple quadrupole mass spectrometer was operated in electron ionization (EI) mode with an ionization voltage of 35 eV, ion source temperature of 230 °C, quadrupole temperature of 150 °C, and transfer line temperature of 300 °C, scanning from *m*/*z* 50 to 500 at 2.5 s per scan, solvent delay 3.75 min. Default instrument settings of collision gas flow of N_2_ at 1.5 mL min^−1^ and quench gas of He at 2.35 mL min^−1^ were used. Agilent MassHunter software was used for acquisition, data handling, and reporting.

### 3.6. Validation Studies

The validation of the analytical methods was implemented according to SANTE 11312/2021 guidelines [27]. Method performance for LC-amenable and GC-amenable residues was verified, including parameters such as linearity, LOQs, recovery, precision, and measurement uncertainties. The validation procedures were extensively described in our previous papers [5,11,13,40,41]. Five levels (0.002, 0.005, 0.01, 0.05, and 0.1 mg kg^−1^) of matrix-matched calibrations were prepared for each target analyte.

For recovery, replicate homogenates (*n* = 5) were spiked at two levels of concentrations: an upper level of fortification of 0.05 mg kg^−1^ and a lower level of sample spiking with residue concentration of 0.01 mg kg^−1^. The repeatability of the method was assessed through the relative standard deviations (RSD_r_, %) associated with measurements of target compounds performed during recovery analyses on the same day. Over a one-week period, the within-laboratory method reproducibility (RSD_R_, %) was assessed. This involved two laboratory analysts performing matrix homogenate spiking, extraction, and analysis on different days. Each operator extracted and analyzed a batch of fortified homogenates (*n* = 10). To determine the expanded measurement uncertainty for each analyte, trueness (bias) and within-laboratory reproducibility uncertainties were taken into account.

## 4. Conclusions

This study has focused on the determination of 511 pesticide residues including widely used pesticides in the citrus industry, non-approved residues, and six highly polar substances in satsuma mandarin samples. Two sample extraction methods, QuEChERS and QuPPe, have been successfully applied for the analysis of non-polar/medium-polar and highly polar substances, respectively. This three-year monitoring study showed that 91.6% of 226 satsuma mandarin samples collected from the Izmir region, Turkey, contained one or multiple residues, up to nine residues. Forty different residues comprising 23 insecticides, 14 fungicides, two acaricides, and one insect growth regulator were detected in mandarin samples during the three harvesting years. While one residue was found in 15.9% of mandarin samples, two or more residues were recorded in 75.7% of samples. In 22.1% of the mandarin samples, the residue concentrations exceeded the MRLs. Among the residues, phosphonic acid (48.7%), spirotetramat (46.5%), fludioxonil (25.2%), pyrimethanil (24.8%), imazalil (24.3%), and 2-phenylphenol (23.5%) were the most frequently found pesticides in satsuma mandarins. The increase in the use of active ingredients in mandarin farming can be attributed to a combination of factors, including the need to manage pests and diseases effectively, meet market demands, improve crop quality, address environmental conditions, adopt sustainable practices, and adhere to regulatory requirements.

These results showed that official citrus monitoring programs should be conducted routinely by governments. Moreover, more strictly controlled measures for hormone-disrupting pesticides such as imazalil should be enacted to protect consumers. The influence of various processing techniques including washing, peeling, and juicing on pesticide residues in mandarins and other citrus fruits should also be investigated. Conducting toxicity studies on the potential synergistic or additive effects of multiple pesticide residues found in citrus fruits will provide valuable information for risk assessment. Furthermore, the cumulative dietary exposure of consumers to detected residues should be analyzed to formulate appropriate risk management measures and establish revised MRLs.

## Figures and Tables

**Table 1:** The presence and quantification of pesticide residues in mandarins in 2019.

Pesticide	Type of Residue	EU MRL (mg kg^−1^)	% of Samples <LOQ	% of Samples between LOQ-MRL	% of Samples >MRL	Range (mg kg^−1^)	
						Min.–Max.	Mean
Acetamiprid	IN	0.9	89.7	10.3	-	0.010–0.129	0.056
Buprofezin	IN	0.01	96.6	-	3.4	0.013	0.013
Carbendazim *	FU	0.7	96.6	3.4	-	0.038	0.038
Chlorpyrifos-methyl *	IN/AC	0.01	96.6	3.4	-	0.010	0.010
Deltamethrin	IN	0.04	96.6	3.4	-	0.015	0.015
Fludioxonil	FU	10	96.6	3.4	-	0.627	0.627
Fluopyram	FU	0.9	96.6	3.4	-	0.013	0.013
Fosetyl **	FU	150	93.1	6.9	-	0.006–0.351	0.179
Imazalil	FU	5	96.6	3.4	-	0.801	0.801
Imidacloprid *	IN	0.9	96.6	3.4	-	0.012	0.012
Lambda-cyhalothrin	IN	0.2	93.1	6.9	-	0.029–0.190	0.110
Malathion	IN	2	65.5	34.5	-	0.014–0.716	0.141
Phosmet *	IN/AC	0.5	96.6	3.4	-	0.014	0.014
Phosphonic acid **	FU	150	27.6	72.4	-	0.028–3.835	0.826
Pirimicarb	IN	3	96.6	3.4	-	0.037	0.037
Propiconazole *	FU	0.01	96.6	-	3.4	1.008	1.008
Pyrimethanil	FU	8	96.6	3.4	-	0.701	0.701
Pyriproxyfen	IN	0.6	96.6	3.4	-	0.028	0.028
Spirotetramat	IN	0.5	82.8	17.2	-	0.017–0.112	0.052
Sulfoxaflor	IN	0.8	82.8	17.2	-	0.010–0.015	0.012
Tau-fluvalinate	IN	0.4	89.7	10.3	-	0.063–0.228	0.135
Thiophanate-methyl *	FU	6	96.6	3.4	-	0.022	0.022

IN: insecticide; FU: fungicide; AC: acaricide; * not approved in the EU; ** sum of fosetyl, phosphonic acid, and their salts expresses as fosetyl.

**Table 2:** The presence and quantification of pesticide residues in mandarins in 2020.

Pesticide	Type of Residue	EU MRL (mg kg^−1^)	% of Samples <LOQ	% of Samples between LOQ-MRL	% of Samples >MRL	Range (mg kg^−1^)	
						Min.–Max.	Mean
2-Phenylphenol	FU	10	55.9	44.1	-	0.584–2.667	0.993
Acetamiprid	IN	0.9	89.2	10.8	-	0.019–0.318	0.067
Azoxystrobin	FU	15	98.9	1.1		0.010	0.010
Buprofezin	IN	0.01	90.3	2.2	7.5	0.010–0.109	0.042
Chlorpyrifos *	IN/AC	0.01	94.6	-	5.4	0.038–0.418	0.149
Cypermethrin	IN	2	98.9	1.1	-	0.512	0.512
Deltamethrin	IN	0.04	97.8	2.2	-	0.012–0.037	0.025
Difenoconazole	FU	0.6	98.9	1.1	-	0.378	0.378
Esfenvalerate	IN	0.02	98.9	1.1	-	0.017	0.017
Fenbutatin-oxide *	AC	0.01	92.5	1.1	6.5	0.010–0.047	0.028
Fludioxonil	FU	10	53.8	46.2	-	0.011–0.648	0.131
Imazalil	FU	5	53.8	46.2	-	0.408–1.006	0.675
Lambda-cyhalothrin	IN	0.2	98.9	1.1	-	0.111	0.111
Malathion	IN	2	82.8	16.1	1.1	0.011–2.855	0.493
Phosphonic acid **	FU	150	41.9	58.1	-	0.039–39.386	2.917
Pirimicarb	IN	3	83.9	16.1	-	0.040–0.165	0.084
Propiconazole *	FU	0.01	91.4	-	8.6	0.020–0.171	0.044
Pyrimethanil	FU	8	53.8	46.2	-	0.329–1.200	0.588
Pyriproxyfen	IN	0.6	89.2	10.8	-	0.021–0.140	0.053
Spirodiclofen *	AC	0.4	93.5	6.5	-	0.010–0.166	0.049
Spirotetramat	IN	0.5	44.1	55.9	-	0.011–0.324	0.061
Sulfoxaflor	IN	0.8	89.2	10.8	-	0.011–0.131	0.040
Tau-fluvalinate	IN	0.4	91.4	8.6	-	0.029–0.385	0.156
Tetraconazole	FU	0.02	97.8	2.2		0.016–0.019	0.018

FU: fungicide; IN: insecticide; AC: acaricide; * not approved in the EU; ** sum of fosetyl, phosphonic acid, and their salts expresses as fosetyl.

**Table 3:** The presence and quantification of pesticide residues in mandarins from 2021.

Pesticide	Type of Residue	EU MRL (mg kg^−1^)	% of Samples <LOQ	% of Samples between LOQ-MRL	% of Samples >MRL	Range (mg kg^−1^)	
						Min.–Max.	Mean
2-Phenylphenol	FU	10	88.5	11.5	-	0.809–1.258	0.979
Acetamiprid	IN	0.9	75.0	25.0	-	0.010–0.121	0.032
Bifenthrin *	IN	0.05	99.0	1.0	-	0.017	0.017
Boscalid	FU	2	99.0	1.0	-	0.012	0.012
Buprofezin	IN	0.01	84.6	-	15.4	0.011–0.164	0.063
Chlorpyrifos *	IN/AC	0.01	98.1	1.0	1.0	0.010–0.013	0.012
Chlorpyrifos-methyl *	IN/AC	0.01	99.0	-	1.0	0.012	0.012
Cyantraniliprole	IN	0.9	97.1	2.9	-	0.010–0.106	0.044
Cypermethrin	IN	2	96.2	3.8	-	0.011–0.024	0.019
Difenoconazole	FU	0.6	92.3	7.7	-	0.010–0.119	0.062
Etoxazole	IN	0.1	97.1	2.9	-	0.013–0.032	0.021
Fenbutatin-oxide *	AC	0.01	95.2	-	4.8	0.013–0.359	0.102
Flonicamid	IN	0.15	99.0	1.0	-	0.018	0.018
Fludioxonil	FU	10	87.5	12.5	-	0.201–0.413	0.313
Fosetyl **	FU	150	94.2	5.8	-	0.110–0.164	0.135
Imazalil	FU	5	89.4	10.6	-	0.436–0.702	0.605
Imidacloprid *	IN	0.9	98.1	1.9	-	0.015–0.029	0.022
Malathion	IN	2	80.8	19.2	-	0.010–1.596	0.256
Novaluron *	IGR	0.01	98.1	-	1.9	0.019–0.111	0.065
Phosphonic acid **	FU	150	66.3	33.7	-	0.026–5.342	1.844
Pirimicarb	IN	3	95.2	4.8	-	0.015–0.182	0.073
Propiconazole *	FU	0.01	93.3	-	6.7	0.031–0.086	0.054
Pyridaben	IN/AC	0.3	86.5	12.5	1.0	0.011–0.318	0.113
Pyrimethanil	FU	8	88.5	11.5	-	0.271–0.601	0.473
Pyriproxyfen	IN	0.6	78.8	21.2	-	0.010–0.166	0.072
Spinosad	IN	0.3	99.0	1.0	-	0.012	0.012
Spirodiclofen *	AC	0.4	85.6	14.4	-	0.018–0.385	0.064
Spirotetramat	IN	0.5	53.8	42.3	3.8	0.010–1.485	0.155
Sulfoxaflor	IN	0.8	93.3	6.7	-	0.012–0.231	0.066
Tau-fluvalinate	IN	0.4	79.8	20.2	-	0.010–0.358	0.090
Thiacloprid *	IN	0.01	98.1	-	1.9	0.013–0.033	0.023
Thiophanate-methyl *	FU	6.0	99.0	1.0	-	0.017	0.017

FU: fungicide; IN: insecticide; AC: acaricide; IGR: insect growth regulator; * not approved in the EU; ** sum of fosetyl, phosphonic acid, and their salts expresses as fosetyl.

## Data Availability

Not applicable.

## Supplementary Materials

The following supporting information can be downloaded at: https://www.mdpi.com/article/10.3390/molecules28145611/s1, Table S1: MRM transitions and in-house validation data for 40 residues.
