# Supplementary material for: Pesticide Residues in Mandarins: Three-Year Monitoring Results
Source: Molecules. 2023 Jul 24;28(14):5611. doi: 10.3390/molecules28145611 (PMC10385200; doi:10.3390/molecules28145611)
Supplement: Supplementary file 1 [file molecules-28-05611-s001.zip › molecules-2531143-supplementary.pdf]

Supplementary material

Table S1. MRM transitions and in-house validation data for 40 residues.

**Pesticide**	**Analyzed by**	**MRM transitions, *m/z* (collision energy, eV)**		**Recovery (%)**		**Repeatability (%RSD_r, *n*=6)**		**Within-laboratory reproducibility (%RSD_R, *n*=12)**		***U* (%)**
		quantification	qualification	0.01 mg kg^{-1}	0.05 mg kg^{-1}	0.01 mg kg^{-1}	0.05 mg kg^{-1}	0.01 mg kg^{-1}	0.05 mg kg^{-1}	
2-Phenylphenol	GC-MS/MS	169.0 → 141.1 (15)	169.0 → 115.1 (25)	101.1	94.4	6.12	2.67	4.47	3.25	15.5
Acetamiprid	LC-MS/MS	223.1 → 126.1 (17)	223.1 → 56.2 (11)	85.6	94.4	1.38	0.48	1.34	2.69	25.4
Azoxystrobin	LC-MS/MS	404.2 → 372.2 (9)	404.2 → 344.2 (23)	92.7	92.8	0.86	0.51	1.74	3.01	19.6
Bifenthrin*	LC-MS/MS	440.2 → 181.1 (7)	440.2 → 166.1 (20)	84.8	103.3	5.01	2.34	3.34	2.65	26.3
Boscalid	LC-MS/MS	345.1 → 309.0 (15)	343.1 → 307.0 (17)	92.9	91.6	1.90	0.90	2.71	3.02	22.7
Buprofezin	LC-MS/MS	306.2 → 201.0 (10)	306.2 → 116.1 (21)	89.6	98.1	0.95	0.63	1.72	2.87	18.4
Carbendazim*	LC-MS/MS	192.1 → 160.1 (15)	192.1 → 132.1 (33)	91.1	92.1	1.18	0.34	0.62	3.29	14.5
Chlorpyrifos*	LC-MS/MS	351.9 → 199.9 (15)	349.9 → 199.7 (15)	103.6	102.8	4.13	0.59	5.64	3.06	14.8
Chlorpyrifos-methyl*	LC-MS/MS	324.0 → 125.0 (13)	322.0 → 125.0 (21)	99.8	98.5	6.17	2.28	8.78	3.49	20.3
Cyantraniliprole	LC-MS/MS	474.9 → 286.1 (10)	472.9 → 284.1 (10)	82.9	93.5	6.43	1.55	5.80	3.66	31.8
Cypermethrin	LC-MS/MS	435.0 → 193.0 (12)	433.0 → 191.0 (12)	96.1	102.4	14.67	8.41	13.56	6.65	36.4
Deltamethrin	LC-MS/MS	522.8 → 505.8 (6)	522.8 → 280.6 (12)	113.5	109.4	3.91	1.58	8.07	2.30	20.5
Difenoconazole	LC-MS/MS	406.2 → 337.1 (13)	406.2 → 251.1 (23)	90.8	96.9	2.32	0.31	1.70	3.45	19.4
Esfenvalarate	LC-MS/MS	439.0 → 169.0 (17)	437.0 → 167.0 (14)	89.2	105.0	16.31	1.80	12.54	1.73	28.0
Etoxazole	LC-MS/MS	360.0 → 141.0 (15)	360.0 → 113.0 (23)	105.4	109.9	0.84	0.65	1.53	2.84	14.9
Fenbutatin-oxide*	LC-MS/MS	519.3 → 351.1 (35)	519.3 → 197.0 (55)	100.1	103.8	3.77	0.56	3.00	2.35	10.4
Flonicamid	LC-MS/MS	230.1 → 203.1 (14)	230.1 → 148.0 (32)	80.4	92.8	3.53	1.12	3.44	2.66	34.5
Fludioxonil	LC-MS/MS	247.0 → 180.0 (33)	247.0 → 126.0 (34)	74.1	93.5	7.16	3.21	3.25	4.28	42.2
Fluopyram	LC-MS/MS	397.0 → 208.0 (20)	397.0 → 173.0 (30)	89.4	94.9	2.41	0.78	2.26	2.77	22.2
Fosetyl	LC-MS/MS	109.0 → 81.0 (12)	109.0 → 63.0 (34)	99.0	86.5	7.61	5.25	7.67	2.39	19.4
Imazalil	LC-MS/MS	297.1 → 159.0 (19)	297.1 → 41.2 (31)	91.6	94.8	0.84	0.76	1.50	2.75	19.7
Imidacloprid*	LC-MS/MS	256.1 → 209.0 (10)	256.1 → 175.0 (12)	85.5	93.2	1.94	0.37	1.19	2.90	25.9
Lambda-cyhalothrin	LC-MS/MS	467.1 → 450.0 (6)	467.1 → 225.0 (14)	107.4	107.0	5.83	2.78	7.73	2.78	19.9
Malathion	LC-MS/MS	330.9 → 285.0 (0)	330.9 → 127.0 (4)	79.7	95.5	6.37	1.07	3.79	3.08	30.8
Novaluron*	LC-MS/MS	492.7 → 158.0 (12)	492.7 → 140.7 (46)	91.6	99.9	3.92	1.59	5.77	2.34	15.8
Phosmet*	LC-MS/MS	317.9 → 160.0 (21)	317.9 → 133.0 (28)	91.8	95.3	0.92	1.14	1.97	2.79	18.9
Phosphonic acid	LC-MS/MS	81.0 → 79.0 (15)	81.0 → 63.0 (35)	101.1	105.8	3.09	3.12	4.66	2.68	12.1
Pirimicarb	LC-MS/MS	239.2 → 182.1 (11)	239.2 → 72.1 (15)	91.5	95.6	1.20	0.56	1.98	2.92	19.2
Propiconazole*	LC-MS/MS	342.2 → 159.0 (27)	342.2 → 69.2 (17)	77.6	92.8	6.64	1.51	5.13	3.38	39.0

Pyridaben	LC-MS/MS	365.2 → 309.1 (7)	365.2 → 147.1 (23)	102.6	104.6	1.42	1.39	1.40	2.61	8.5
Pyrimethanil	LC-MS/MS	200.0 → 107.0 (26)	200.0 → 82.0 (28)	90.6	95.9	0.66	0.82	4.97	2.67	22.9
Pyriproxyfen	LC-MS/MS	322.2 → 185.0 (19)	322.2 → 96.1 (11)	97.5	106.5	1.58	0.77	1.81	2.62	8.8
Spinosad	LC-MS/MS	732.5 → 142.1 (35)	732.5 → 98.0 (55)	102.4	98.6	0.70	0.67	1.52	2.13	10.4
Spirodiclofen*	LC-MS/MS	411.0 → 313.0 (11)	411.0 → 71.0 (16)	98.6	99.2	1.11	0.27	9.28	3.89	29.5
Spirotetramat	LC-MS/MS	374.1 → 330.1 (21)	374.1 → 302.1 (23)	89.9	91.3	1.29	0.73	2.03	2.48	24.5
Sulfoxaflor	LC-MS/MS	277.9 → 174.0 (2)	277.9 → 154.0 (29)	89.0	95.3	1.45	0.97	1.68	3.11	21.9
Tau-fluvalinate	LC-MS/MS	503.1 → 208.1 (15)	503.1 → 181.1 (25)	102.6	108.9	1.42	2.43	4.69	3.28	15.3
Tetraconazole	LC-MS/MS	372.0 → 159.0 (35)	372.0 → 70.0 (20)	73.9	93.6	4.54	1.01	7.61	3.03	42.8
Thiacloprid*	LC-MS/MS	253.0 → 126.0 (16)	253.0 → 90.0 (35)	91.1	93.9	1.23	0.52	1.16	2.73	20.2
Thiophanate-methyl*	LC-MS/MS	343.0 → 151.0 (20)	343.0 → 93.0 (56)	105.8	97.8	0.20	0.30	5.54	2.44	15.1

*Not approved in the EU.
